# Editorial: Developing next-generation therapeutics to defeat mutation-driven cancer

**DOI:** 10.3389/fimmu.2024.1487952

**Published:** 2024-09-26

**Authors:** Frances Chow, François Lamoureux, Gatien Moriceau

**Affiliations:** ^1^ Norris Comprehensive Cancer Center, University of Southern California, Los Angeles, CA, United States; ^2^ CRCI2NA/INSERM UMR 1307/CNRS UMR 6075/Nantes Université, Nantes, France; ^3^ Division of Dermatology, Department of Medicine, University of California, Los Angeles, Los Angeles, CA, United States

**Keywords:** next generation therapeutics, immunotherapy, antibody drug conjugate (ADC), targeted therapy, drug resistance, combination therapy, cancer

Mutation-driven cancers are one of the major medical challenges of the current century. Over the past decade, intense comprehensive sequencing has revealed the genomic, transcriptomic, and epigenetic landscapes in cancer, leading to the investigation of novel tailored therapeutic approaches. Mutation-induced hyperactivated signaling plays a significant role in cancer and has risen to recognition as a potential druggable target via the exploitation of cancer cell vulnerabilities. Despite revolutionary advances in immunotherapies and targeted therapies, innate and acquired resistance develop nearly universally, highlighting the need for ongoing research in innovative therapeutic strategies to defeat mutation-driven cancers.

The aim of the present Research Topic is to feature: (i) preclinical proof of concept studies to support well-defined single or combination regimens, (ii) target-based mechanisms that lend themselves to combination therapies by conserving or boosting immune responses, and (iii) approaches to overcome acquired resistance to current therapies.

As editors of this Research Topic, we had the pleasure of reading highly innovative manuscripts. We summarize the main findings and perspectives of each accepted manuscript in this editorial. This Research Topic features one original research manuscript and three reviews.

The original research manuscript by Yang et al. explores the possibility of predicting pancreatic ductal adenocarcinoma (PDAC) prognosis based on KRAS mutation status. They combined a multi-omics approach using two different cohorts to stratify overall survival risk and identify key components of ferroptosis-associated pathways as potential druggable targets. Their model lays the foundation for next-generation therapeutic strategies against PDAC.

The review by Song et al. comprehensively and concisely describes the potential role of the transcription factor AP-1, which they propose may offer a multi-pronged attack against cancer by enhancing targeted- and immune-based approaches. In addition to regulating the transcription of downstream target genes, AP-1 targeted inhibition can reduce immune checkpoint expression, overcome CAR-T cell exhaustion, overcome resistance to HDAC targeted therapy, and sensitize to PARP1, CDK4/6 and EGFR targeted therapy.

The review by Hsu et al. offers insight into the exploration of antibody-drug conjugates (ADCs) for MET, HER3, EGFR, Trop2, and other targets in EGFR-mutated non-small cell lung cancer (NSCLC). The first EGFR-targeted drug approved by the FDA in 2013, osimertinib, a third-generation tyrosine kinase inhibitor, is now used for advanced NSCLC. However, resistance to osimertinib occurs, leading to consideration of ADCs as new therapeutic strategy. The authors describe therapeutic perspectives that could overcome resistance and present several key clinical trials which may extend this promising therapeutic approach to malignancies beyond NSCLC.

The review by Benjamin et al. elegantly presents the evolution of FGFR-targeting therapy clinical trials for metastatic urothelial carcinoma. The lessons learned from these trials of monotherapy erdafitinib, the combination of erdafitinib plus chemotherapy, and the combination of erdafitinib plus immunotherapy help to inform ongoing and future planned clinical trials of FGFR-targeting therapies. The authors propose unique approaches to overcome acquired resistance by targeting the PI3K pathway, the V561M mutation within the FGFR1 tyrosine kinase domain, and the EGFR pathway.

The guest Editors would like to thank all authors for contributing their highly stimulating results in this topic and all the Reviewers for giving their time to make this Research Topic possible.

